# Exploring the transcriptional cooperation between RUNX2 and its associated elncRNA RAIN

**DOI:** 10.1038/s41419-024-07058-x

**Published:** 2024-09-14

**Authors:** Emanuele Vitale, Veronica Manicardi, Mila Gugnoni, Federica Torricelli, Benedetta Donati, Silvia Muccioli, Elisa Salviato, Teresa Rossi, Gloria Manzotti, Simonetta Piana, Alessia Ciarrocchi

**Affiliations:** 1 Laboratory of Translational Research, Azienda Unità Sanitaria Locale-IRCCS di Reggio Emilia, Reggio Emilia, Italy; 2 Clinical and Experimental Medicine PhD Program, University of Modena and Reggio Emilia, Modena, Italy; 3 Pathology Unit, Azienda Unità Sanitaria Locale-IRCCS di Reggio Emilia, Reggio Emilia, Italy

**Keywords:** Long non-coding RNAs, Transcriptomics

## Abstract

Recent insights into the mechanisms controlling gene expression identified enhancer-associated long non-coding RNAs (elncRNAs) as master players of transcription in cancers. RUNX2, a mammalian RUNT-related transcription factor, is increasingly recognized in cancer biology for its role in supporting survival and progression also in thyroid cancer (TC). We recently identified, within the RUNX2 locus, a novel elncRNA that we named RAIN (RUNX2 associated intergenic lncRNA). We showed that RAIN and RUNX2 expression correlate in TC, both in vitro and in vivo, and that RAIN promotes RUNX2 expression by interacting with and affecting the activity of the RUNX2 P2 promoter through two distinct mechanisms. Here, we took forward these observations to explore the genome-wide transcriptional function of RAIN and its contribution to the RUNX2-dependent gene expression program in TC. By combining multiple omics data, we demonstrated that RAIN functionally cooperates with RUNX2 to the regulation of a subset of functionally related genes involved in promoting matrix remodeling, migration, and loss of differentiation. We showed that RAIN interacts with RUNX2 and its expression is required for the efficient recruitment of this TF to its target regulatory regions. In addition, our data revealed that besides RUNX2, RAIN governs a hierarchically organized complex transcriptional program by controlling a core of cancer-associated TFs that, in turn, orchestrate the expression of downstream genes. This evidence indicates that the functional cooperation observed between RAIN and RUNX2 can be a diffuse work mechanism for this elncRNA.

## Introduction

Cancer is a complex and dynamic system that changes in time and space adapting to the contextual challenges to survive and progress [1, 2]. This plasticity is sustained by the precise execution of specific gene expression programs which, in turn, are guaranteed by the rapid and highly performant rewiring of the transcriptional network along the genome [3]. This requires the functional integration of many interspersed regulatory elements, and it is executed by the assembly on chromatin of large complexes within which multiple intra- and inter-molecular interactions are established [4, 5].

Central in these dynamics are Transcription Factors (TFs) that serve as primary docking sites for the entire transcriptional machinery [3, 6]. For their centrality in the execution of vital cellular processes, cancer-associated TFs are currently regarded as potential vulnerabilities of cancer cells [7, 8]. RUNX2 is a Runt-related TF required during embryogenesis for the development of skeletal tissue and the morphogenesis of other organs, including the thyroid gland [9, 10]. Aberrantly reactivated during oncogenesis, this TF plays a fundamental role in many tumors including thyroid cancer (TC) [11, 12]. In this context, RUNX2 supports cancer cells’ proliferation, migration, invasiveness, and metastatic spreading. Indeed, RUNX2 promotes metastatization in a dual manner. On one hand, it partakes to the execution of the TGF-β-induced Epithelial to Mesenchymal Transition (EMT) process. On the other hand, it favors cancer cell osteomimicry, a transdifferentiating process required for metastasis bone colonization. Above all, the aberrant expression of this TF is essential to strength cancer cell resistance to stress and death [12–17].

Enhancer-associated long non-coding RNAs (elncRNAs) are a class of long non-coding transcripts expressed from active enhancers (ENHs). By binding DNA, they affect chromatin organization and function regulating the transcription of either neighboring or distant genes. The current hypothesis is that these elncRNAs represent a further and finer layer of ENH regulatory activity [18, 19]. Due to their ability of establishing multiple and dynamic interactions with DNA, proteins, and other RNA molecules, elncRNAs are emerging as essential scaffolds of the multiunit complexes that control gene expression. Besides, their high tissue- and context-specificity and their ability of working in a non-stoichiometric manner make these molecules ideal candidates to fine tune cell specification, both in physiological and pathological conditions [20, 21]. Despite the raising interest in their functions, several aspects of the elncRNAs biology still remain superficial and far from being fully comprehended.

Recently, we described RAIN as a novel elncRNA associated with RUNX2 [14, 22]. We showed that RAIN has two alternative TSSs located within two transcriptionally active RUNX2 distal ENHs. Its expression correlates with the activity of the ENHs and the expression of RUNX2 both in TC cell lines and patients’ tissues. Besides, RAIN stimulates *in cis* the expression of RUNX2, controlling the RUNX2 P2 promoter by at least two diverse mechanisms. By associating with its core component WD Repeat Domain 5 (WDR5), RAIN promotes the recruitment of the Set/MLL histone methyltransferase complex to the P2 promoter, favoring its activation and fostering RNA-PolII recruitment. In parallel, by restraining Negative Elongation Factor Complex Member E (NELFe) binding downstream to the RUNX2 TSS, RAIN facilitates the RNA–Polymerase II (RNA-PolII) transition into active elongation, sustaining the robust expression of RUNX2 [22]. In addition, by performing in vitro experiments we demonstrated that RAIN promotes oncogenic features of cancer cells, influencing cell proliferation and migration [22].

In this study, we aimed to extend the functional characterization of RAIN by exploring its pleiotropic impact on the transcriptional landscape of TC and its potential contribution to the RUNX2-dependent gene expression program. We showed that, besides controlling its expression, RAIN cooperates with RUNX2 in regulating a subset of common target genes. RAIN facilitates RUNX2 recruitment to target regulatory regions, thereby supporting its transcriptional activity. Furthermore, our findings reveal that RUNX2 is not the sole TF under RAIN regulation. Rather, this elncRNA plays a direct role in controlling a subset of cancer-associated TFs that contribute to defining the overall RAIN transcriptional program. These results indicate the existence of a hierarchically organized transcriptional cascade in which this elncRNA controls and cooperates with cancer-promoting TFs, including RUNX2.

## Methods

### Cell culture

Human Papillary Thyroid Carcinoma (PTC) TPC1 cells were obtained from Prof. Massimo Santoro (University of Naples, Naples, Italy) and cultured in DMEM-Glutamax (Gibco, Thermo Fisher Scientific, Waltham, MA, USA) supplemented with 10% Fetal Bovine Serum (FBS) and 1% penicillin-streptomycin (Gibco, Thermo Fisher Scientific Waltham, MA, USA). Metastatic PTC MDA-T41 cells were purchased from American Type Culture Collection (ATCC, Manassas, VA, USA) and grown in RPMI-1640 (EuroClone S.p.A., Milan, Italy) supplemented with 1% non-essential amino acids (Gibco, Thermo Fisher Scientific Waltham, MA, USA), 10% FBS and 1% penicillin-streptomycin. All cell lines were grown at 37 °C/5% CO_2_ and routinely tested for mycoplasma infection. Cells were authenticated by SNP profiling at Multiplexion GmbH (Heidelberg, Germany) in 2023.

### CRISPR-Interference (CRISPRi)

The Genome Browser online platform was used to identify PAM sequences and design single-guide RNAs (sgRNAs). RAIN and non-targeting (NT) sgRNAs (Supplementary Table 1) were purchased from Eurofins Genomics (Milan, Italy) and cloned in the lentiviral vector Plv-hU6-sgRNA hUbC-dCas9-KRAB-T2a-Puro (Addgene plasmid #71236; a gift from Charles Gersbac) with Esp3I (Thermo Fisher Scientific, Waltham, MA, USA). Lentiviral particles were produced as previously described [23]. HEK293T were co-transfected with the sgRNA-dCas9-Krab-expressing vector, the packaging plasmids pRSV-Rev and pMDLg/pRRE, and the envelope plasmid pMD2.G (AddGene #12253, #12251, and #12259, gift from Didier Trono) using Lipofectamine 2000 (Thermo Fisher Scientific Waltham, MA, USA). Viral supernatants were collected 48 h after the transfection, filtered with 0.45 µm filters, and added with 2 µg/mL polybrene. Infected cells were selected with 1 µg/mL puromycin (Merck Millipore, Burlington, MA, USA) for 5 days.

### RNA extraction and quantitative real-time PCR (qRT-PCR)

Total RNA was extracted Maxwell® RSC simply RNA Cells (Promega, Madison, Wisconsin, USA) or RNeasy Plus Mini Kit (QIAGEN, Germantown, MD, USA) and retrotranscribed with iScript cDNA kit (Biorad, Hercules, CA, USA) following the manufacturer’s instruction. qRT-PCR was performed using Sso Fast EvaGreen Super Mix (Biorad, Hercules, CA, USA) in the CFX96 Real-Time PCR Detection System (Biorad, Hercules, California, USA). Relative expression was calculated using the 2^−ΔΔCt^ method by normalizing to the reference genes Actin B (ACTB), and Cyclophilin A (CYPA). Sequences of qRT-PCR primers are listed in Supplementary Table 2.

### Western Blot

Total proteins were extracted using Passive Lysis Buffer (Promega, Madison, Wisconsin, USA) supplemented with Protease-Inhibitor (PI) cocktail (Roche, Basel, Switzerland). Lysates were loaded on Mini-Protean TGX pre-cast gels (Biorad, Hercules, CA, USA) and SDS-PAGE was performed using the Biorad apparatus (Biorad, Hercules, CA, USA). Membranes were incubated with 1:1000 of RUNX2 (#D1L7F, Cell Signaling Technology) and 1:5000 β-ACTIN (#A1978, Sigma Aldrich) primary antibodies. Immunoblot detection was performed with the appropriate HRP-conjugated secondary antibodies (GE Healthcare, Piscataway, NJ, USA) and Clarity Western ECL substrate (Bio-Rad, Hercules, CA, USA).

### Chromatin isolation by RNA-purification sequencing assay (ChIRP-seq)

ChIRP assay was conducted as described by Chu et al. [24]. 20 RAIN-antisense oligonucleotides were designed with the Biosearch Technologies online ChIRP Probe Designer (Biosearch Technologies Hoddesdon, UK). RAIN-probes with 3’ BiotinTEG were purchased from Eurofins Genomics (Milan, Italy).

ChIRP was performed starting from a total amount of 80 × 10^6^ TPC1. Cells were crosslinked with 1% glutaraldehyde (CARLO ERBA Reagents S.r.l., Milan, Italy) in PBS for 10 min at room temperature. Glutaraldehyde was quenched with 0.125 M glycine incubated for 5 min at room temperature. Crosslinked cells were lysed with ChIRP Lysis Buffer (50 mM TRIS-HCl pH7, 10 mM EDTA, 1% SDS) supplemented with 0.1 U/µl Superase-In (Thermo Fisher Scientific, Waltham, MA, USA) and Protease Inhibitors (PI) (Roche, Basel, Switzerland). Lysed cells were sonicated with Bioruptor Pico Sonicator (Diagenode, Denville, NJ, USA) to obtain 200-500 bp chromatin fragments. 1% of ChIRP volume was kept as DNA/RNA input. Half of the sonicated volume was treated with 100 µg/ml RNaseA and 0.1 U/ml RNaseH (ChIRP negative control). RNase-treated and untreated samples were diluted 1:3 with ChIRP Hybridization Buffer (750 mM NaCl, 1 mM EDTA, 1% SDS, 15% Formamide, supplemented with 0.1 U/ml Superase-In and Protease Inhibitors). RAIN pull-down was carried out with two independent probe sets (EVEN and ODD) (Supplementary Table 3). After probe hybridization (37 °C, overnight) and precipitation with Streptavidin Magnetic Beads (Thermo Fisher Scientific, Waltham, MA, USA) (37 °C, 2 h), samples were washed 5 times with ChIRP Wash Buffer (2X SSC, 20% SDS). In the last wash, beads were divided for RNA and DNA purification.

#### RNA purification

Ten percent of ChIRP volume was used to purify RNA. Dry beads and input were resuspended in Proteinase K buffer (10 mM TRIS HCl pH7, 1 mM EDTA, 0.5% SDS, 100 mM NaCl) and treated with 100 µg Proteinase K (Promega, Madison, WI, USA) for 45 min at 50 °C. Samples were boiled at 95 °C for 10 min and then RNA was extracted with miRNeasy Kit (Qiagen, Germantown, MD, USA) following manufacturer's instructions. RNA was retrotranscribed into cDNA and RAIN enrichment was evaluated in qRT-PCR. Each qRT-PCR value was normalized over the appropriate Input and reported in graphs as % Input.

#### DNA purification

Ninety percent of ChIRP volume was used to extract DNA. Dry beads were resuspended in 150 µl of DNA-elution Buffer (500 mM NaHCO3, 1% SDS, 100 µg/ml RNaseA, and 0.1 U/ml RNaseH) and incubated at 37 °C for 30 min while shacking. Beads were precipitated on a magnetic rack and a second round of elution was performed. The two eluates were combined (final volume = 300 µl). 300 µl of DNA-elution Buffer was added to the Input and incubated for 30 min at 37 °C while shacking. Eluted DNA samples were incubated at 50 °C for 45 min with 300 µg Proteinase K. DNA was purified with PCR Purification Kit (Qiagen, Hilden, Germany) following the manufacturer’s instructions. Absolute DNA quantity was established using the ProNex DNA QC Assay (Promega, Madison, WI, USA) in the Real-Time CFX96 system (Biorad, Hercules, CA, USA). ChIRP-libraries were prepared with a starting DNA quantity of 150 pg with ThruPLEX DNA-Seq Kit (Takara Bio Ink, Kusatsu, Japan), and two independent experiments were run on Illumina NextSeq500 high-output cartridge (single-stranded, read length 75 bp-1 × 75).

### Chromatin immunoprecipitation assay (ChIP)

ChIP assays were conducted following the manufacturer’s instructions with SympleChIP Enzymatic Chromatin IP Kit with Magnetic Beads Kit (Cell Signaling, Danvers, Massachusetts, USA). Briefly, 8 × 10^6^ cells/IP were crosslinked with 1% formaldehyde for 10 min. Chromatin was fragmented with 1 µl MNase for 20 min at 37 °C. Immunoprecipitations were performed with 2 µg of anti-RUNX2 (#D1L7F, Cell Signaling Technology) or 2 µg of Rabbit IgG antibodies (#2729, Cell Signaling Technologies) and 30 µl of magnetic beads. 2% of IP chromatin volume was kept as Input.

RUNX2 relative enrichment on selected regions was assessed by qRT-PCR. Each qRT-PCR value was normalized over the appropriate input control. Relative fold enrichment of RAIN-KD over control cells was calculated for each target and represented on the graph.

### RNA immunoprecipitation assay (RIP)

RIP assays were performed as previously described [25]. 10-15 × 10^6^ cells/IP were recollected and crosslinked with 1% formaldehyde for 15 min at room temperature. Crosslink was quenched with 0.125 M glycine. Crosslinked cell pellets were resuspended in Nuclei Isolation Buffer (5X concentration: 1.28 M sucrose, 40 mM Tris-HCl pH 7.5, 20 mM, MgCl2, 4% Triton X-100) supplemented with PI. The nuclear pellet was resuspended in RIP buffer (150 mM KCl, 25 mM Tris-HCl pH 7.5, 5 mM EDTA, 0.5 mM DTT, 0.5% NP40) supplemented with PI and Superase-In. Nuclear lysate was sonicated using Bioruptor Pico Sonicator (Diagenode, Denville, NJ, USA). 10% of IP volume was kept as input. Immunoprecipitation was carried out with 4 µg of anti-RUNX2 (#D1L7F, Cell Signaling Technology) or 4 µg of Rabbit IgG (#2729, Cell Signaling Technologies) antibodies overnight at 4 °C on a wheel. Protein-antibody complexes were precipitated with Dynabeads Protein G magnetic beads (Thermo Fisher Scientific, Waltham, MA, USA). RNA was purified with miRNeasy Kit (Qiagen, Germantown, MD, USA) following manufacturer instructions. RIP-RNA was retrotranscribed into cDNA and RAIN enrichment was evaluated in qRT-PCR. Each qRT-PCR value was normalized over the appropriate input control. RAIN enrichment was calculated as a fold of RUNX2-IP over control IgG.

### RNA-Sequencing (RNA-seq)

RNA-sequencing libraries were obtained starting from 100 ng of total RNA following Illumina Stranded TotalRNA PrepLigation with Ribo-zero Plus protocol (Illumina, San Diego, CA, USA). Sequencing was performed using Illumina NEXTSeq high-output cartridge (double-stranded, read length 75 bp-2 × 75 cycles).

### Patients cohort and ethical approval

Formalin-fixed paraffin-embedded (FFPE) samples of a retrospective cohort of PTC patients were collected at the Pathology Unit of Azienda USL-IRCCS of Reggio Emilia, Italy. Twenty-four metastatic PTC (DM) and 24 non-metastatic PTC (CTRL) samples retrieved between 2010 and 2021 were selected. Clinical features of the selected PTC samples are shown in Supplementary Table 4. Each patient who participated in the study provided written informed consent for the biological studies. This study was authorized by the local Ethics Committee (Comitato Etico dell’Area Vast Emilia Nord; protocol no 2017/0071406) and conducted according to Helsinki declaration.

### Nanostring nCounter analysis

RAIN expression was evaluated on the selected cohort of PTC patients with the Nanostring nCounter system (Nanostring Technologies, Seattle, Washington, USA) using a custom panel. Total RNA was extracted by Maxwell® RSC RNA FFPE kit (Promega, Madison, Wisconsin, USA) starting from 5 slides of 5 µm FFPE tissue. RNA quantity and quality were assessed by NanoDrop 2000 (Thermo Fisher Scientific, Waltham, MA, USA). Only samples that reached the required RNA standard quality (A260/A280 ≥ 1.7 and A260/A230 ≥ 1.8) were analyzed. Analysis of detected gene counts was performed by nSolver Analysis Software 4.0 (Nanostring Technologies, Seattle, WA, USA). For samples that passed imaging quality controls (24 CTRL and 22 DM), raw gene counts were normalized on technical controls and housekeeping genes with the lowest coefficient of variation.

Fold Changes were calculated as ratio between the expression profile of DM and CTRL samples. For each comparison, *p*-value (as a two-tailed Student’s *t*-test) and False Discovery Rate (FDR) obtained by the Benjamini-Hochberg method, were calculated.

Bioinformatics analyses on gene expression program (GEP) were conducted by R Software v4.0.3.

### Public dataset analysis

RNA-seq FASTQ files of the cohort of 27 Normal thyroid tissue, 30 Primary and 29 Metastatic thyroid cancer lesions published by Sanghi A. et al. (GEO accession number GSE162515) [26] were downloaded through SRAtoolkit. Paired reads were mapped to the human reference genome GRCh38.p14 using STAR (v2.7.11) with default parameters. Transcripts were counted at a gene level using RSEM (v1.3.1) on Gencode release 44 modified to include RAIN locus. RAIN isoforms coordinates were retrieved from UCSC Genome Browser (GRCh37/hg19), converted using UCSC LiftOver tool then manually added to Gencode v44 primary assembly .gtf file.

### Bioinformatics analysis

#### RNA-seq

Sequencing data quality was assessed using the FastQC v0.11.8 software [27]. High-quality paired-end reads were aligned to the human reference genome (GRCh38 patch 13) and genes were annotated on Gencode release 30. RSEM [28] was applied on paired FASTQ files to map counts through STAR (v2.7.1) [29] and estimate transcript abundance. Differential analysis was performed with the R package DESeq2 v1.32.0 [30]. Genes with low reads were excluded and a False Discovery Rate (FDR) of 0.1 was adopted as a significance threshold. Gene Ontology Biological Process (GO-BP) and Reactome Pathway enrichment analyses were performed with the enrichR package, considering a significance threshold of 0.05 for the *p*-value adjusted according to the Benjamini-Hochberg correction (adjusted *p*-value).

#### ChIRP-seq

Sequencing data quality was assessed using the FastQC v0.11.8 software [27]. Residual adapter sequences were removed using Trimmomatic (v.0.39) software [31]. Filtered reads were aligned to the human reference genome (GRCh38/hg38 assembly) by using Bowtie2 v2.3.5.1 [32]. Duplicated and unmapped reads were removed with Picard tool (available online at http://broadinstitute.github.io/picard) and Samtools v1.9 [33]. Peak calling was performed using MACS2 (v.2.1.3.3) [34] with default parameters and considering a cut-off of q < 0.05. RAIN peaks (.bed) identified separately with the EVEN and ODD probe sets were intersected with Bedtools v2.30 [35] and only the overlapping regions were retained and considered as true RAIN-bound genomic elements. ChIPseeker R package (v 1.28.3) [36] was used for assigning peaks to the nearest genes, according to GRCh38/hg38 annotation, using a TSS window of ±3 kb.

#### FIMO motif search

To predict the TF binding on RAIN targets, DNA sequences were extracted from a window of 1.5 kb upstream and 0.5 kb downstream of RAIN targets TSSs. TFs motifs were identified by FIMO [37] using HOCOMOCO and JASPAR as reference databases. q-value < 0.05 was adopted as a significance cut-off for motif enrichment.

#### Pathway enrichment and similarity

All functional enrichment analyses were performed by enrichR R package using Gene Ontology - Biological Process (GO-BP) as reference database. Pathway similarity was calculated on shared genes using Jaccard method, then *cluster_terms* function from simplifyEnrichment R package was applied to cluster GO-BPs on their similarity score by Louvain clustering algorithm.

R version 4.1.0 was used for bioinformatics analyses.

### Statistical analysis

Statistical analysis was performed using GraphPad Prism Software (version 9.3.0 for Windows, GraphPad Software, San Diego, CA, USA). Statistical significance was determined using the Student’s t-test (two-tailed, unpaired). Each experiment was replicated two to five times.

## Results

### Characterization of the RAIN transcriptional program in primary and metastatic TC cell lines

**Fig. 1:**
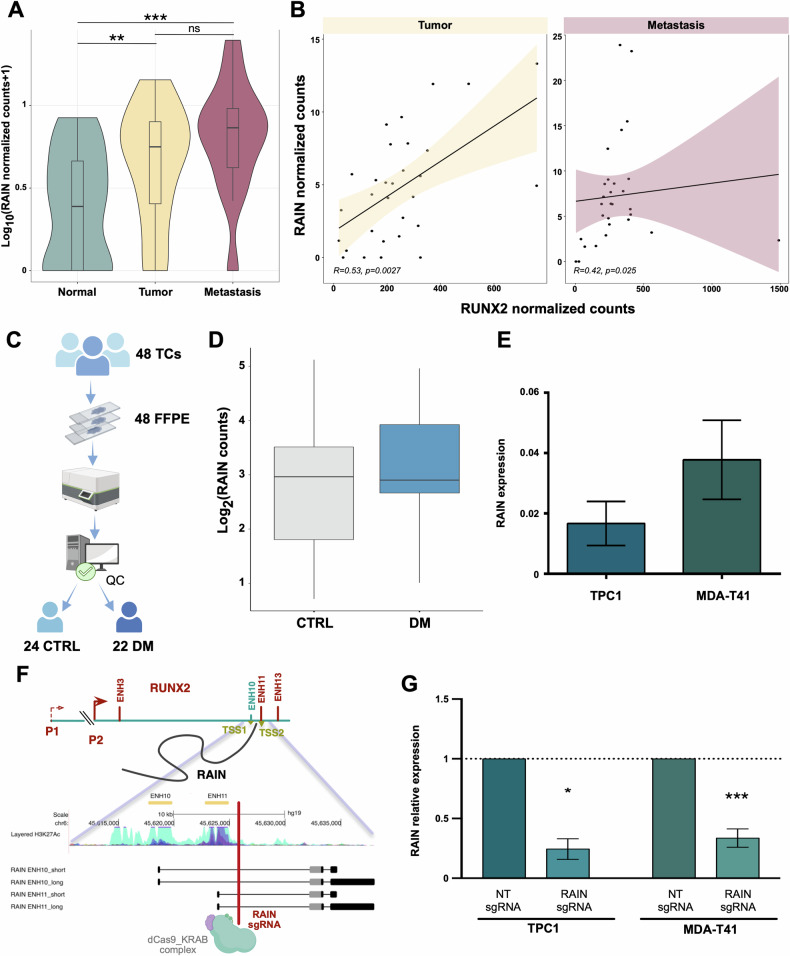
RAIN expression correlates with TC metastasis. **A** Violin plot showing RAIN normalized counts in normal thyroid, primary tumors, and metastases from 34 TC patients. **p-value < 0.01, ***p-value < 0.001 **B** Scatterplots showing the Spearman correlation analysis of RUNX2 and RAIN expressions in primary tumor and metastatic TC datasets. The correlation coefficient (R) and *p-*value (*p*) of each analysis are shown. In each graph, lines represent linear regression and colored areas show confidence intervals. **C** Pipeline of samples analysis by nCounter Nanostring technology. **D** Boxplot of RAIN expression level in patients’ cohort. CTRL=control samples, DM=metastatic samples. **E** Histogram of RAIN expression level in TPC1 and MDA-T41 cell lines assessed by qRT-PCR. **F** Graphical representation of CRISPRi strategy designed for RAIN knock-down. **G** Assessment of RAIN knock-down by qRT-PCR. Bars represent mean±s.d. of RAIN expression relative to control (NTsgRNA) of two to three replicates. *p-value < 0.05, ***p-value < 0.001.

Our previous work highlighted the contribution of RAIN in supporting TC metastatic progression by influencing cell proliferation, migration, and invasion [14, 22]. To consolidate these data, we examined the expression of RAIN by analyzing publicly available RNA-seq data from 34 TC patients. In line with its role in promoting TC aggressiveness, RAIN expression was higher in metastatic lesions as compared to primary tumors (Fig. 1A). This difference was even greater when considering matched primary and metastatic samples from the same patient (log2FC = 1.07, *p*-value < 0.05) (Supplementary Fig. 1A). Moreover, according to our previous findings, we observed a significant correlation between RAIN and RUNX2 expression levels in both primary tumor (Spearman correlation (*R*) = 0.53, *p* = 0.003) and metastatic samples (Spearman correlation (*R*) = 0.42, *p* = 0.025) (Fig. 1B). Finally, we explored RAIN expression in a retrospective cohort of 48 PTCs with different clinical behaviors by Nanostring nCounter technology [38, 39]. This cohort included primary tumors from 24 PTCs that developed distant metastasis (DM) and 24 PTCs that did not present metastatic behavior (CTRL) with a minimum follow-up of 10 years (Fig. 1C). After quality checks, the expression profile of 24 CTRL and 22 DM was further analyzed. The expression of RAIN tended to be higher and more heterogeneous in the DM cohort even if this trend was not significant (Fig. 1D).

To get insight into the mechanism through which RAIN contributes to TC aggressiveness, we selected TPC1 and MDA-T41 as primary and metastatic PTC cell models [40] (Fig. 1E). We used a CRISPR-interference (CRISPRi) approach to stably repress RAIN expression in TC cell lines. Since RAIN TSSs reside within two RUNX2-ENHs, we designed sgRNAs targeting the RAIN gene body outside the RUNX2-ENHs, avoiding a direct perturbation of these regulatory elements (Fig. 1F, G).

**Fig. 2:**
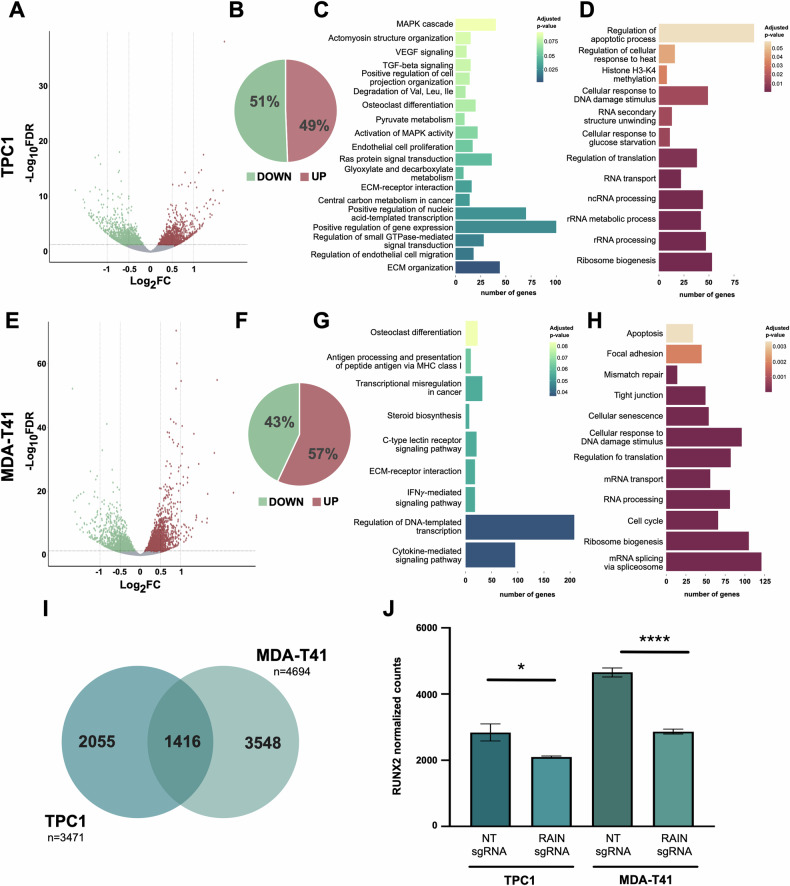
RAIN silencing alters TC cells transcriptional program. **A, E** Volcano plots of DEGs in TPC1 (**A**) and MDA-T41 (**E**). **B, F** Pie charts of the percentage of the significantly up- and downregulated genes in TPC1 (**B**) and MDA-T41 (**F**). **C, G** Functional enrichment on downregulated genes in TPC1 (**C**) and MDA-T41 (**G**). **D, H** Bar length is proportional to the number of genes belonging to each category. Functional enrichment on upregulated genes in TPC1 (**D**) and MDA-T41 (**H**). Bar length is proportional to the number of genes belonging to each category. **I** Venn diagram of RAIN DEGs common and exclusive to TPC1 and MDA-T41. **J** Assessment of RUNX2 expression change upon RAIN KD by RNA-seq. Bars represent mean±s.d. of RUNX2 normalized counts in RAIN KD (RAINsgRNA) cells versus control (NTsgRNA) cells. * adjusted *p*-value < 0.05, **** adjusted *p*-value < 0.0001.

RNA-seq profiling followed by differential analysis comparing RAIN KD (RAINsgRNA) and control (NTsgRNA) cells showed a wide effect of RAIN loss on TC transcriptome. 3471 genes were found to be significantly deregulated in TPC1 (Fig. 2A). Of these 1714 (49.38%) were upregulated and 1757 (50.62%) were downregulated (Fig. 2B). Downregulated genes were enriched, among the others, in major cancer-associated processes that included VEGF and TGF-β signaling pathways, the MAPK cascade, extracellular matrix (ECM) organization and interactions (Fig. 2C). Conversely, regulation of apoptosis and RNA metabolism were among the top scoring GO categories enriched in genes upregulated following RAIN KD (Fig. 2D).

Upon RAIN KD 4694 genes were found to be significantly altered in MDA-T41 (Fig. 2E). Of these, 2673 were up (56.95%) and 2021 (43.08%) were downregulated (Fig. 2F). GO analysis on downregulated genes confirmed ECM interaction among the top scoring categories and highlighted several immune-related categories as processes under the regulation of this lncRNA (Fig. 2G). By contrast, apoptosis, RNA metabolism, response to DNA damage and cell-cell interaction were among the processes enriched on upregulated genes (Fig. 2H).

1416 genes were found to be commonly deregulated in TPC1 and MDA-T41 (Fig. 2I). As expected, RUNX2 resulted significantly downregulated in both cell lines (Fig. 2J), as confirmed by western blot analysis (Supplementary Fig. 1B). Of note, the extent of RUNX2 downregulation in this analysis was comparable with the one we previously observed silencing RAIN by LNA-Gapmers [22]. This suggests that the CRISPRi design does not perturb the activity of the RUNX2 ENHs.

### Mapping RAIN genomic landscape in TC cells

**Fig. 3:**
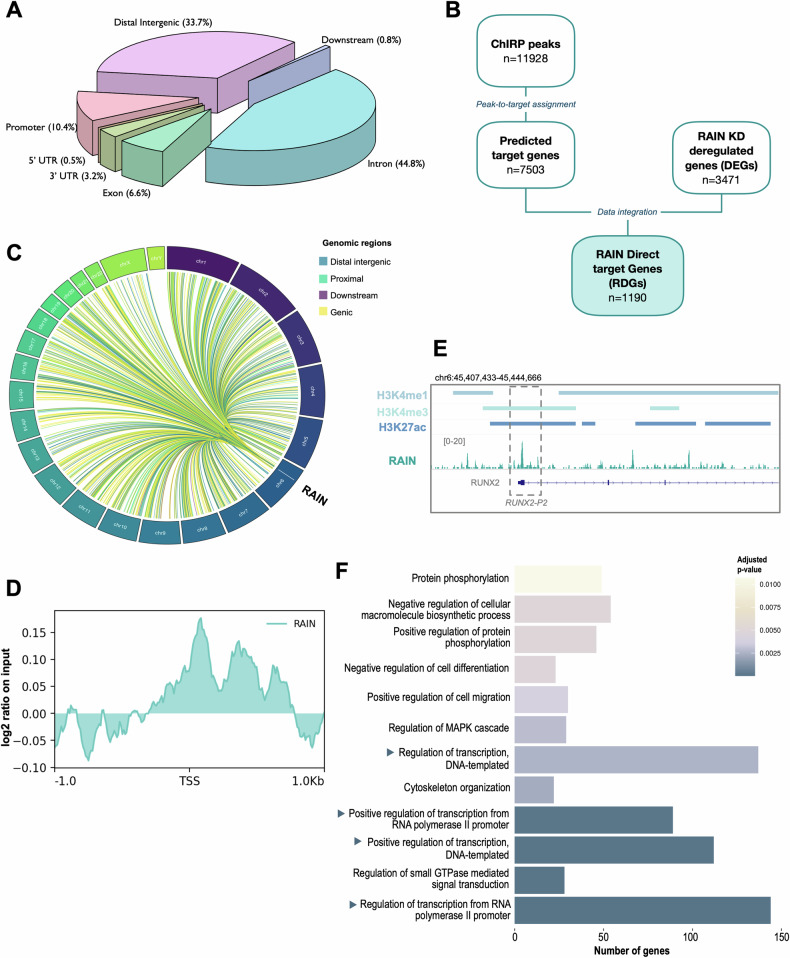
Identification of RAIN genomic binding sites in TC cells. **A** Peak-to-target assignment of RAIN peaks. Pie chart shows the RAIN peaks distribution on different genomic regions. **B** Flowchart of ChIRP-seq and RNA-seq data integration to retrieve RAIN direct target genes (RDGs). **C** Chord diagram of RDGs distribution on chromosome. Linkers are colored according to the type of genomic element bound by RAIN on its RDG loci. **D** Metaprofile of RAIN signal distribution around RDG-TSSs. **E** Representative ChIRP track showing the binding of RAIN to RUNX2-P2 promoter. RAIN coverage (.bam) was represented together with H3K4me1, H3K4me3, and H3K27ac peaks defined by MACS (.bed). **F** GO biological process (BP) enrichment on RDGs. Bar length is proportional to the number of genes belonging to each BP. BPs related to gene expression regulation are highlighted.

To better understand the transcriptional function of RAIN we performed ChIRP-seq analyses to get a map of the RAIN genomic distribution in TPC1 (Supplementary Fig. 1C–E). We identified 11928 RAIN peaks that were assigned to the nearest gene to annotate their genomic localization and predict target genes under their regulation. This analysis showed that RAIN preferentially locates within gene bodies (44.77% introns, 6.57% exons, 3.75% 5′-3′ UTRs). 33.68% of RAIN peaks were assigned to distal intergenic elements and 10.43% localized at the level of promoters (Fig. 3A). The list of RAIN-putative targets was intersected with RNA-seq data to filter out all predicted genes whose expression was not perturbed by RAIN loss (Fig. 3B). This analysis identified 1190 RAIN-Direct target Genes (RDGs), which represent 34.28% of RAIN KD deregulated genes. Genes whose expression was affected by RAIN-KD and did not display RAIN binding on their putative regulatory regions were defined RAIN-indirect target Genes (RIGs) (*n* = 2281, 65.72% of DEGs).

RDGs were distributed across the genome and located in different chromosomes, underlining that RAIN can affect transcription in trans (Fig. 3C). At RDG sites, RAIN resulted to be preferentially associated with intragenic, distal intergenic and promoter regions (Fig. 3C). As shown in Fig. 3D RAIN signal accumulates at TSS. In accordance with our previous data, RAIN was found to bind the RUNX2-P2 promoter, in a region enriched in both H3K27ac and H3K4me3 (Fig. 3E).

GO-BP enrichment analysis on RDGs is displayed in Fig. 3F. Most of the top-scoring enriched BPs are involved in transcriptional regulation, suggesting that RAIN main role is to modulate the expression of genes involved in gene expression regulation. The widespread gene expression dysregulation observed upon RAIN loss is in line with these findings. Moreover, only a small fraction of RAIN DEGs are directly regulated by this elncRNA, while most of them are altered by indirect effects.

Altogether these results suggest that RAIN global transcriptional program is largely ascribable to RDGs.

### A core of cancer-related TFs mediates RAIN transcriptional program

**Fig. 4:**
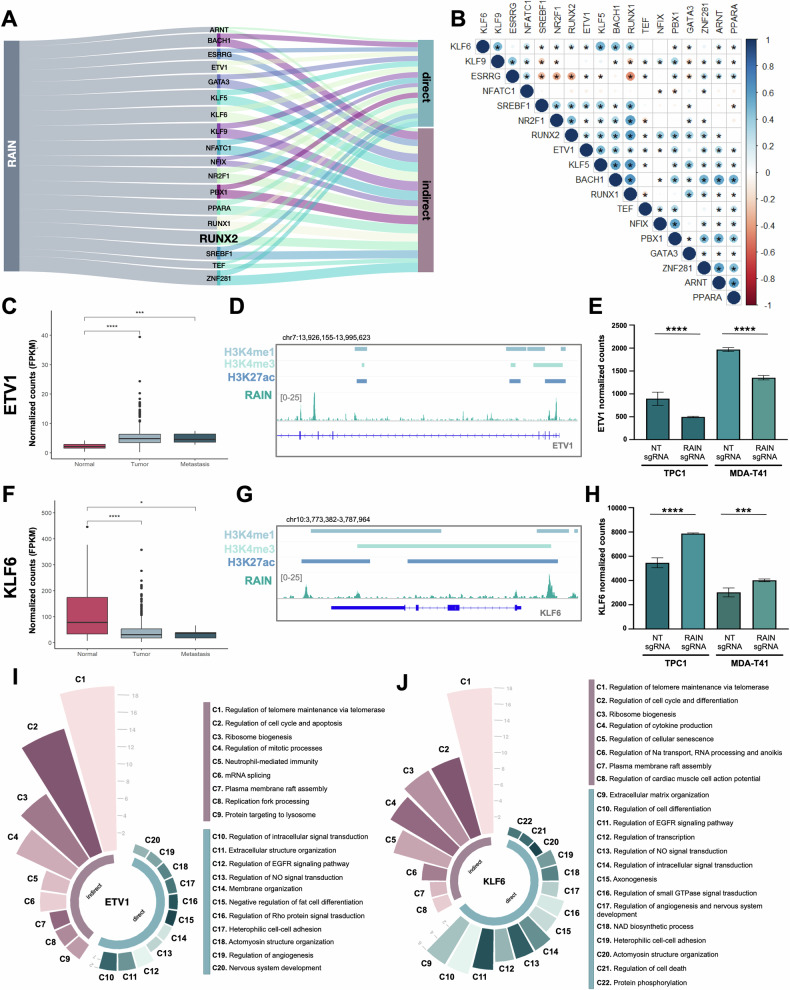
RDG-TFs cooperate with RAIN in defining its downstream gene expression program. **A** Alluvial plot highlighting the 18 RDG-TFs identified by FIMO and their putative RDG (direct) and RIG (indirect) target genes. **B** Correlation matrix of the 18 RDG-TFs identified by FIMO in PTC patients (THCA-TCGA). In red inversely correlated, in blue directly correlated genes. * indicates significance, dot size and color are proportional to the correlation coefficient. **C**, **F**. Boxplot of ETV1 (**C**) and KLF6 (**F**) expression level in normal, tumor, and metastatic tissues from THCA-TCGA patients. *p-value < 0.05; ***p-value < 0.001; ****p-value < 0.0001. **D**, **G**. Representative ChIRP track showing the binding of RAIN to ETV1 (**D**) and KLF6 (**G**) genomic loci. RAIN coverage (.bam) was represented together with H3K4me1, H3K4me3, and H3K27ac peaks defined by MACS (.bed). **E**, **H**. Assessment of ETV1 (**E**) and KLF6 (**H**) expression change upon RAIN KD by RNA-seq. Bars represent mean±s.d. of ETV1 or KLF6 normalized counts in RAIN KD (RAINsgRNA) cells versus control (NTsgRNA) cells. ***adjusted *p*-value < 0.001, ****adjusted *p*-value < 0.0001. **I, J** GO-BP enrichment analysis on RDGs and RIGs predicted as ETV1 (**I**) and KLF6 (**J**) targets. Circular barplots show the groups of GO-BPs clustered on gene similarity. Bar length is proportional to the number of BPs included in each cluster. Legend on the right indicates the name assigned to each cluster.

In accordance with GO-BP enrichment analysis, 112 TFs and 82 transcription cooperators were identified as RDGs in TPC1. Of these, 33 TFs and 21 co-factors were confirmed to be coherently affected by RAIN KD also in MDA-T41 (Supplementary Fig. 2A). RUNX2 was included in the final list. We named these RAIN target TFs as RDG-TFs. Intrigued by this observation, we hypothesized that RAIN transcriptional activity in TC could be structured according to a hierarchical organization in which the RDG-TFs represent the first layer and serve to coordinate the expression of the other downstream RAIN targets (either RDGs or RIGs). To validate this hypothesis, we used the FIMO algorithm to assess the putative enrichment of these TFs at promoters of the 1416 RAIN DEGs common to TPC1 and MDA-T41 (Supplementary Fig. 2A). Alluvial plot visualizes the results of this analysis. 18 RDG-TFs were predicted to regulate both RIGs and RDGs (Fig. 4A). These data suggest that RDG-TFs may contribute to the RAIN gene program by propagating RAIN regulatory effect on RIGs expression and by directly cooperating with RAIN in the transcriptional control of RDGs (Fig. 4A).

We took advantage of the THCA-TCGA dataset to explore these 18 RDG-TFs expression in vivo. Noticeably, we observed that these TFs showed a diffuse significant correlation among them in tumor patients, which support their belonging to a common transcriptional program (Fig. 4B). No information about RAIN was available in this dataset. However, RUNX2, that we demonstrated to be strongly positively correlated to RAIN in human TC samples [22], displayed a significant correlation with almost all the other analyzed RDG-TFs (14 out of 17) supporting the validity of our analysis. Besides, we analyzed the expression of these 18 RDG-TFs in normal, primary, and metastatic tissues (Fig. 4C, F and Supplementary Fig. 2B). Twelve out of 18 TFs displayed differences during disease progression, underlying their potential involvement in TC progression. As RUNX2, many of these TFs have already been reported to support cancer aggressiveness in other cancers [41–46]. We selected ETV1 and KLF6 as representative RDG-TFs. ETV1 expression was directly positively regulated by RAIN (Fig. 4D, E) and its expression was found to be upregulated in primary tumors and metastatic lesions as compared to the normal counterparts (Fig. 4C), thus suggesting a pro-tumorigenic role of this TF in TC. By contrast, KLF6 displayed the opposite trend, being negatively regulated by RAIN and downregulated in pathological tissues (Fig. 4F–H). Diagrams in Fig. 4I, J show how these TFs mediate RAIN transcriptional function impacting on specific biological categories, including processes linked to cytoskeleton remodeling, cell signaling, angiogenesis, regulation of cell adhesion, telomeres biology, and cellular senescence. Overall, these data support a model in which RAIN directly regulates a core of TFs that, in turn, cooperate with this elncRNA and act on independent genes, triggering a regulatory cascade that culminates in the definition of a complex gene program supporting TC aggressiveness.

### RUNX2 and RAIN functionally cooperate to control metastasis-associated gene expression

**Fig. 5:**
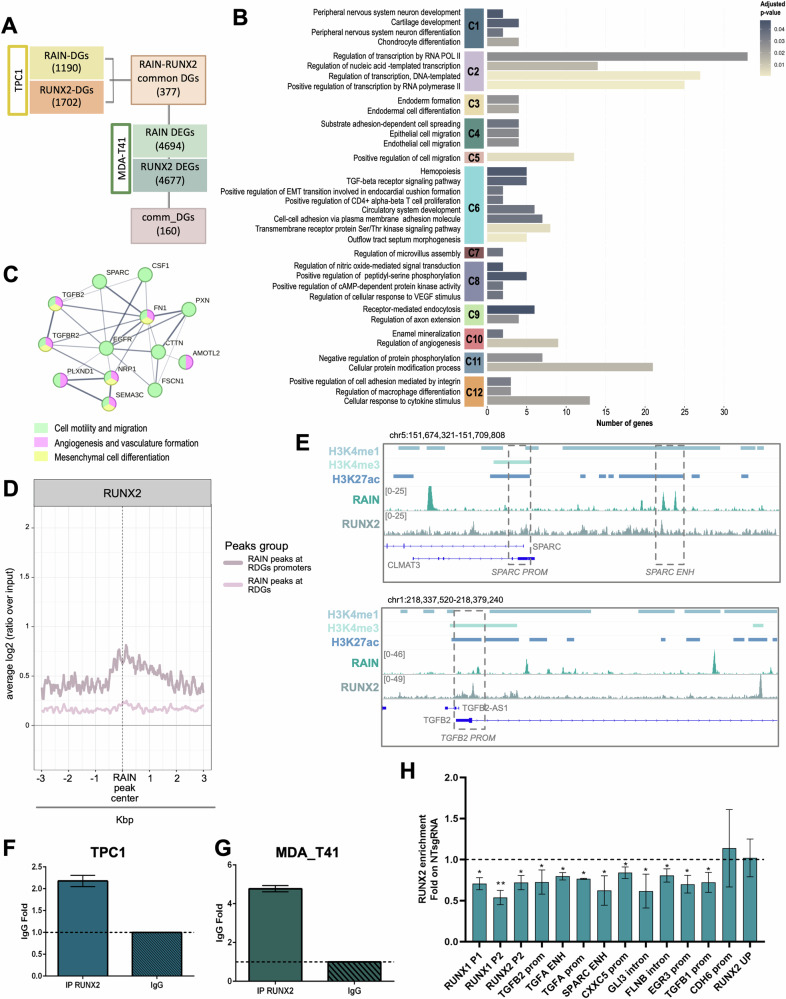
RUNX2 and RAIN functional interplay. **A** RAIN ChIRP-seq, RUNX2 ChIP-seq, and RNA-seq data integration. Flowchart shows the steps of gene lists intersection followed to retrieve the core of genes regulated by both RAIN and RUNX2 in TPC1 and MDA-T41. **B** Bar plot of the GO-BP enrichment analysis on the 160 comm_DGs. Bar length is proportional to the number of genes in each BP. Colored boxes on the left represent the groups of GO-BPs clustered on gene similarity. **C** Protein-protein interaction network of representative RUNX2-RAIN common targets. Gene nodes are colored on the biological process they belong to. **D** Metaprofile showing the RUNX2 enrichment in a 6 kb window around the center of RAIN peaks. Light pink=RUNX2 signal at all RAIN peaks associated with RDGs genomic loci. Dark pink = RUNX2 signal at all RAIN peaks associated with RDGs promoters. **E** Representative ChIRP track showing the binding of RAIN to the regulatory elements of two representative RUNX2-RAIN common targets (SPARC, TGFB2). RAIN and RUNX2 coverage (.bam) were represented together with H3K4me1, H3K4me3, and H3K27ac peaks defined by MACS (.bed). **F, G** qRT-PCR of RUNX2-RIP experiments performed in TPC1 (**F**) and MDA-T41 (**G**). RAIN fold enrichment over IgG (negative control) is represented in the histograms. Graphs show the mean±s.d. of two replicates. **H** qRT-PCR of the RUNX2 ChIP conducted on RAIN-KD and control (NTsgRNA) TPC1. Fold Change enrichment of RUNX2-KD vs control is represented in the graph. Bars show the mean ± s.d. of four to five replicates. Negative control regions = CDH6 prom and RUNX2 UP. *p-value < 0.05; **p-value < 0.001.

RUNX2 was found among the 18 RDG-TFs identified by the FIMO analysis. We predicted 1270 RAIN DEGs, of which 478 RDGs and 792 RIGs, as potential RUNX2 targets. Since we previously characterized the RUNX2 transcriptional landscape in TC [40], we selected RUNX2 as representative RDG-TF to validate our hierarchical model. Starting from TPC1 data, we intersected the lists of RUNX2 direct target genes (RXDGs, *n* = 1702) and RDGs (*n* = 1190) obtaining 377 common direct targets. We further filtered this list on RAIN DEGs (*n* = 4694) and RUNX2 DEGs (*n* = 4677) from MDA-T41 datasets and obtained a list of 160 common direct targets (comm_DGs) shared by both cell lines (Fig. 5A).

Noticeably, GO analysis indicated that these genes were enriched in processes related to regulation of cell migration and differentiation, TGF-β signaling, and angiogenesis (Fig. 5B, C). All these features have already been reported as a central part of the pro-tumorigenic function of RUNX2 in cancer [15]. Noticeably, these biological processes included many genes already known to be key RUNX2 targets in TC and other cancer settings.

Taking advantage of TPC1 datasets, we analyzed the distribution of RUNX2 signal around the RAIN peaks assigned to RDGs. Metaprofile showed the enrichment of RUNX2 signal in the proximity of the center of RAIN-peaks associated with RDG-promoters, suggesting that the two factors are located close to each other on these genomic regulatory elements (Fig. 5D). Figure 5E shows the co-binding of RUNX2 and RAIN on the regulatory regions of two common targets, SPARC, in which both RUNX2 and RAIN associate with a regulatory element displaying chromatin features of active ENH (SPARC ENH), and TGFB2, of which RUNX2 and RAIN co-occupy the promoter region (TGFB2 PROM).

We performed RIP analysis to assess whether RUNX2 interacts with RAIN within the same complex, observing a significant interaction between these two factors in both cell models (Fig. 5F, G).

Finally, we performed RUNX2-ChIP analysis in RAIN-KD and control (NTsgRNA) TPC1 to assess the effect of this elncRNA on the RUNX2 occupancy on the regulatory regions of common targets. All the comm_DGs investigated showed a significant reduction in the binding of RUNX2 on their regulatory regions upon RAIN loss (Fig. 5H). CDH6 was previously described as RUNX2 target [15] but was excluded from comm_DGs. We used CDH6 promoter region as negative control for this assay. Since no significant changes were observed in this region, we concluded that the effect was specific for comm_DGs regulatory regions.

Together these data indicate that RAIN cooperates with RUNX2 in controlling a precise subset of target genes by fostering its recruitment on these common target promoters.

## Discussion

RUNX2 is a cancer-related TF. Aberrantly reactivated during oncogenesis, RUNX2 contributes to cancer aggressiveness in many settings by regulating multiple aspects of cancer cell biology. In TC, RUNX2 has been linked to a pro-metastatic phenotype [47]. We and others reported that RUNX2 controls the expression of genes involved in cellular architecture, cell-cell and cell-matrix adhesion, and extracellular matrix remodeling, coordinating the structural reorganization and conferring properties of movement and invasiveness to TC cells [15–17, 48, 49]. Moreover, in TC and other epithelial tumors, RUNX2 mediates the TGF-β signaling during the EMT process, regulating genes that confer high metastatic potential to these tumors, such as CDH6 [15, 49, 50]. Finally, RUNX2, which is known as the master of skeletal development, has been reported to support metastasis bone colonization by promoting the transdifferentiation of epithelial cancer cells toward a bone like phenotype [13, 47, 48, 51–54].

Conversely, very little is still known about the molecular mechanisms through which this TF works to coordinate these biological functions at a transcriptional level.

In this work, we partially addressed this issue by exploring for the first time the functional cooperation between RUNX2 and RAIN, its associated elncRNA. We provided new and relevant insights not only on the way this TF works but also on how elncRNAs substantially contribute to the definition of multidimensional transcriptional programs laying behind cancer progression.

We demonstrated that RAIN, besides controlling RUNX2 transcription, cooperates with it in the regulation of a common core of direct targets. Functionally, these common targets partake in processes linked to cellular architectural remodeling and differentiation that converge on the definition of a metastatic phenotype. Mechanistically, we showed that RAIN binds to the regulatory elements of these genes within the same regions as RUNX2. Moreover, RAIN silencing causes the displacement of this TF from these regions, while leaving unaffected other regulatory loci on which RUNX2 acts in a RAIN-independent manner. This indicates that RAIN expression reinforces the transcriptional activity of RUNX2 by enhancing its binding on these co-occupied regions. RUNX2-RAIN common targets are enriched in key processes of the RUNX2 gene program in TC progression, thus strengthening the idea that RAIN represents a further and finer level of their expression regulation.

As the annotation and functional characterization of lncRNAs is progressing, it is becoming evident that a complex crosstalk between lncRNAs and TFs exists, especially in physiological and pathological contexts characterized by high transcriptional plasticity, like cancer. The interplay happens at many levels following different mechanisms. In many cases, TFs have been shown to control transcription of specific elncRNAs that in turn cooperate with them to directly regulate their common targets [18, 55]. In this regard, the functional crosstalk between RUNX2 and RAIN appears to be quite original.

In our model, the regulatory cascade starts with RAIN boosting RUNX2 expression, then promoting its transcriptional function on relevant target genes. Named for what they are not, lncRNAs represent one of the most complex and promising entities of our genome. Among them, elncRNAs are emerging as key players in the regulation of gene expression. Most of the functionally characterized elncRNAs have been studied for their *in cis* effect as their activity were limited to a one-on-one action on neighboring genes [18, 56]. However, genome is a complex tridimensional entity and transcription is widely affected by the chromatin folding that place in contact regions far apart in the linear sequence. As the experimental evidence accumulates, the contribution of elncRNAs to the definition of this complex organization is coming into light, revealing a pervasive and pleiotropic action of these transcripts at the genome level. In light of this, a paradigm shift has become necessary in the approach to studying elncRNAs that allows us to take into consideration and characterize the transversal effects of their action at the transcriptional level.

**Fig. 6:**
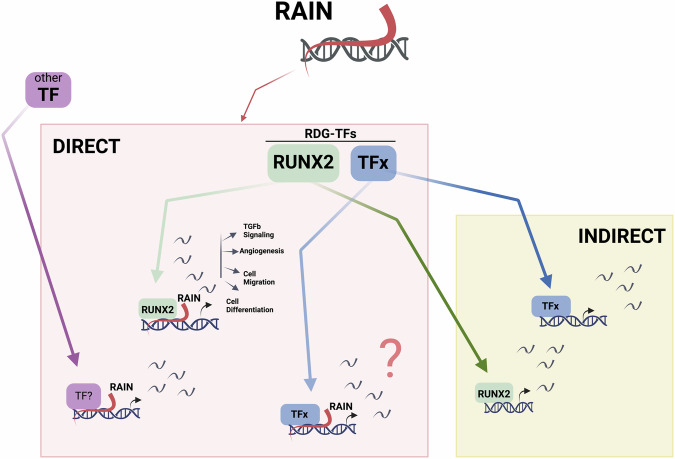
Graphical model of RAIN transcriptional network. Pink box: RAIN direct gene program including RDG-TFs and RDGs controlled by RAIN through its cooperation with either RDG-TFs (RUNX2, TFx) or other TFs (TF?). Yellow box: RAIN indirect gene program.

The data presented in this work strongly support this perspective demonstrating that RAIN does not simply control its nearby protein-coding gene, but governs a complex transcriptional network hierarchically organized. In addition to RUNX2, RAIN directly controls other cancer-associated TFs, that in turn propagate its regulatory function by modulating the expression of other downstream genes. This widespread mechanism of action is supported by the observation that the overall impact of RAIN KD on TC transcriptome significantly overcomes that of RUNX2. Of note, we observed that some of these TFs may play as direct regulators of subgroups of RDGs suggesting their functional cooperation with RAIN. Based on our data, we may suppose that the dual interplay (regulation and cooperation) observed between RAIN and RUNX2 can occur also with other RAIN target TFs and may serve to coordinate specific subsets of functionally related genes, representing a second level of regulation of RAIN on its targets (Fig. 6).

In our previous work, we showed that RAIN controls RUNX2 expression [22]. In this work, we add a further layer to RAIN function showing that this elncRNA is required for an efficient recruitment of TFs on target regulatory elements. The integration of all these functions should result in the robust and buffered expression of genes important for cancer progression. Indeed, in line with this supportive function, we demonstrated that RAIN (even more than RUNX2) correlates with aggressiveness in human TC patients, further supporting the robustness of the proposed model and the biological relevance of this elncRNA.

It is now consolidated that there are many ways through which elncRNAs cooperate to transcription regulation affecting several aspects of the process, from chromatin structure and organization to RNA-PolII progression along the gene body and post-transcriptional processing [18, 56]. In this work, we consolidated the centrality of these molecules in cancer biology demonstrating that RAIN, through multimodal activity, cooperates with RUNX2 and potentially with other TFs to orchestrate the transcriptional program supporting TC progression. Our data, together with accumulating evidence on elncRNAs genomic functions, strongly encourage the systematic characterization of these transcripts to better define their implication in cancer biology.

## Supplementary information

SUPPLEMENTARY FILES

Original Data

## Data Availability

NGS sequencing data generated and analyzed during the current study are available in the Array Express repository. RNAseq experiments: E-MTAB-11049, E-MTAB-13777. ChIP-seq against RUNX2: E-MTAB-11051. ChIRP-seq: E-MTAB-13776.

## Code availability

The code will be available upon reasonable request.
