# Supplementary material for: Exploring the transcriptional cooperation between RUNX2 and its associated elncRNA RAIN
Source: Cell Death Dis. 2024 Sep 14;15(9):673. doi: 10.1038/s41419-024-07058-x (PMC11399121; doi:10.1038/s41419-024-07058-x)
Supplement: Supplementary file 1 — SUPPLEMENTARY FILES [file 41419_2024_7058_MOESM1_ESM.pdf]

Supplementary FIGURE 1

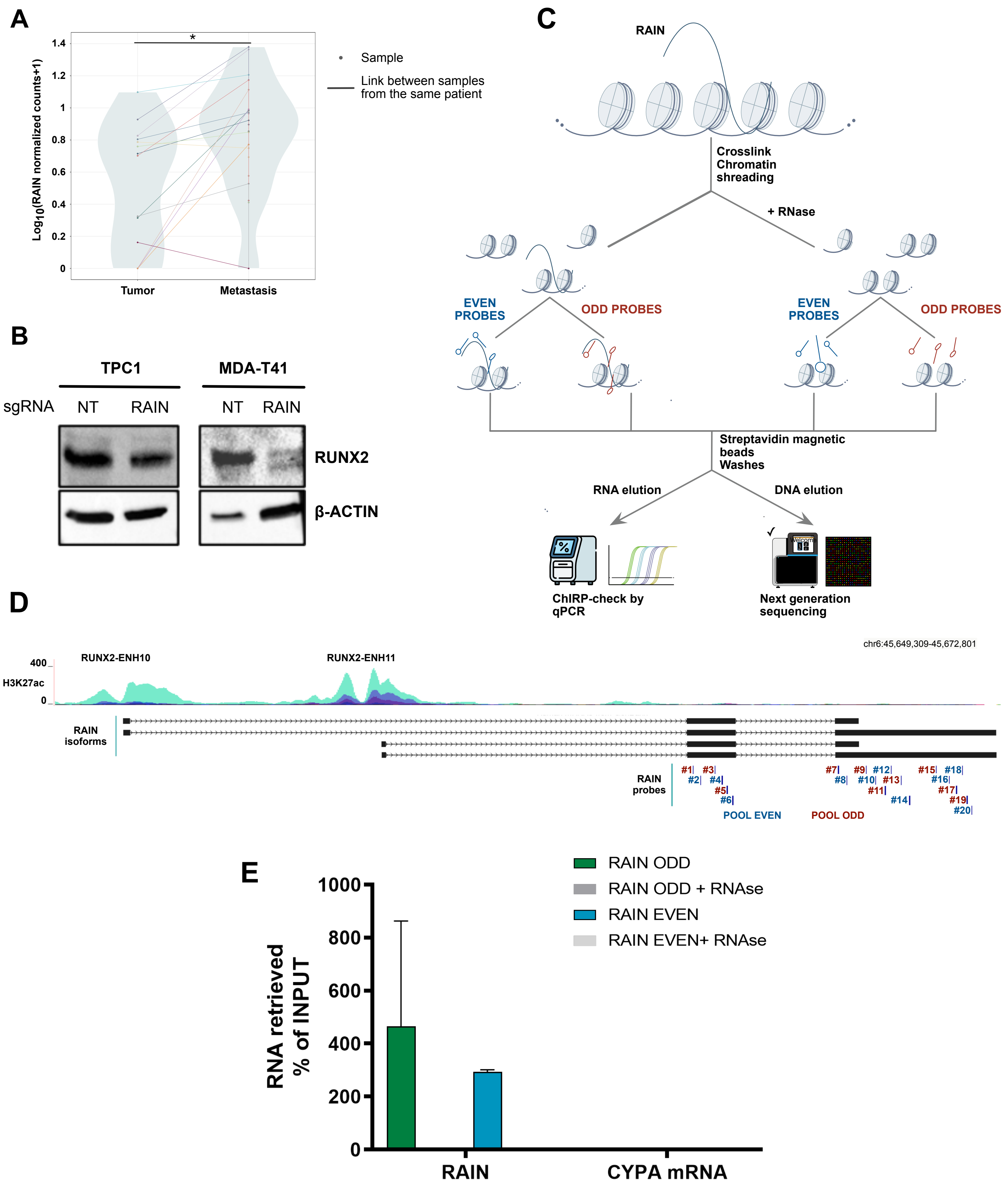

Supplementary FIGURE 2

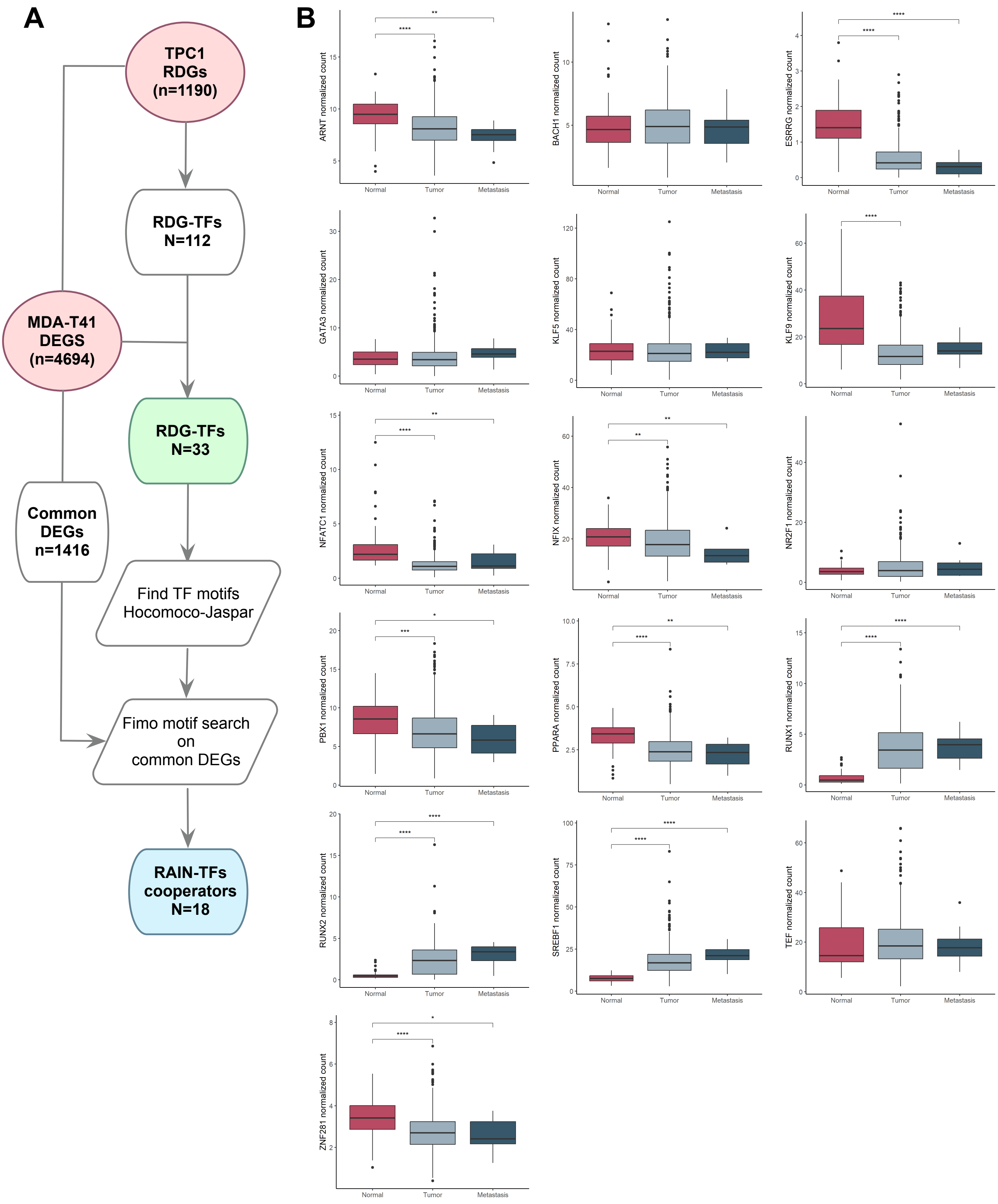

Supplementary Figure Legends

Supplementary Figure S1 A. Violin plot showing RAIN expression in matched primary tumors, and metastases from 14 patients included in the publicly available cohort. Colored dots represent the samples. Colored lines link samples from the same patients. *p-value<0.05 **B.** Western Blot showing RUNX2 protein level in RAIN KD (RAINsgRNA) and control (NTsgRNA) cells. **C.** Schematic overview of the ChIRP-seq approach. **D.** Screenshot of RAIN locus from Genome Browser. Track represents H3K27ac signal at RAIN promoters in different cell lines, genomic regions of RUNX2 ENH10 and ENH11 are highlighted on top of H3K27ac track. All RAIN isoforms and genomic mapping of ChIRP probes are shown. **E.** Control of ChIRP experiment in qRT-PCR. CYPA mRNA was used as negative control.

Supplementary Figure S2 A. Schematic workflow of FIMO analysis. **B.** Boxplots showing the expression levels of 16 out of 18 RAIN-TFs putative cooperators in THCA-TCGA dataset. Gene expression was evaluated by comparing Normal tissue with Primary Tumor and Metastatic lesions. *p-value<0.05; ** p-value<0.01, ***p-value<0.001; ****p-value<0.0001.

Supplementary Table 1 CRISPRi sgRNA sequences

Name	Target	Sequence
RAINsgRNA	RAIN gene body	GCAATACTCCCTCCTGCCAT
NTsgRNA	-	CTGAAAAAGGAAGGAGTTGA

Supplementary Table 2 List of qPCR primers

Target	Forward primer	Reverse primer
Primers for gene expression		
RUNX2	GCTCTTCTTACTGAGAGTGGAAGG	GTGCCTAGGCGCATTTCA
RAIN	CTCAAAGCAAGTCGCCAAAG	CCTGTGATCTGCCCTTTAGC
ACTB	ACCTTCTACAATGAGCTGCG	CCTGGATAGCAACGTACATGG
CYPA	GACCCAACACAAATGGTTCC	TTTCACTTTGCCAAACACCA
Primers for ChIP		
RUNX1-P1	CTGTGGGTTGGTGATGCTC	AGCCTGGCAGTGTCAGAAGT
RUNX1-P2	GGCTGGTCCTCTGGTTTGTT	TGGCTGCCTTCCGAAGAGT
RUNX2-P2	ACCATGGTGGAGATCATCG	GGCAGGGTCTTGTTGCAG
TGFB2 prom	TAGACATGCCGCCCTTCTTC	TGCTGCGGTTTTGAGAGCTC
TGFA prom	GACCATTTTACGGGCGGGC	CCCTGCCTAGTCTGCGTCTT
TGFA ENH	CCTGTGGCATTTGGAGGGTG	TGAATCCCTGTCTGGCCACT
SPARC ENH	CACTGAACGGCTCGCTTCC	GGCGGAGATGATTTCACACCA
CXXC5 prom	TACCCAAGCCTCCCTAAGCC	GCCGTCCCAGACCCTAAAAC
GLI3 intron	GCAGGGCTTTGAAAACGCAC	TAGTAACCCAGCTTTGCGGT
FLNB intron	CCCCACATACTCCAACAAGCA	CTTGGGTATGCAGTGACGCT
EGR3 prom	GGTCGAGGATGTGCGGTTTT	GGGGATTCAGTGCACGTGTT
TGFB1 prom	TGTTGTACAGGGCGAGCAC	CAGATCCTGTCCAAGCTGCG
CDH6 prom	GGCTCCGTTGGCTATGCATA	GGGCTCTCTCTCTGATTGGC
RUNX2 UP	TCTCAAGGTGCCTGTCTGC	TGAAGTTTGGCCTCTGGTCT

Supplementary Table 3 List of RAIN probes used for ChIRP

Name	Sequence	Pool
**RAIN ChIRP_probe1**	ACCTGGGGAAAAGGCCTGAG	ODD
**RAIN ChIRP_probe2**	AGAGATGGTCGAAGGGCAAC	EVEN
**RAIN ChIRP_probe3**	CCATTAGTTGGCATCCGAAA	ODD
**RAIN ChIRP_probe4**	GTCTGTTTCACGCTGAAACG	EVEN
**RAIN ChIRP_probe5**	TTTTTCTGCTAGACGCTCAA	ODD
**RAIN ChIRP_probe6**	CTTTGGCGACTTGCTTTGAG	EVEN
**RAIN ChIRP_probe7**	TTTAGTTGATGCCGGTTTTG	ODD
**RAIN ChIRP_probe8**	TAAGCTACCTAGATGGGTTC	EVEN
**RAIN ChIRP_probe9**	AAGCCATAACAGCCCTAAAG	ODD
**RAIN ChIRP_probe10**	TACACCATGTGAGTGACCAT	EVEN
**RAIN ChIRP_probe11**	AATCACACCGATTCTGGTTT	ODD
**RAIN ChIRP_probe12**	GTTGTGACAGTGCTATTGAC	EVEN
**RAIN ChIRP_probe13**	CTTTGACCCACAGTACTACT	ODD
**RAIN ChIRP_probe14**	CATAGACTGAGAGCCAAGGG	EVEN
**RAIN ChIRP_probe15**	TTGGTAAAGGAAGACGGCCA	ODD
**RAIN ChIRP_probe16**	TGCTACCAAGAGGAAGTCTA	EVEN
**RAIN ChIRP_probe17**	TTGGTAAGGTTCCTTGTTTT	ODD
**RAIN ChIRP_probe18**	AGAGCCATCACGTTGGACAG	EVEN
**RAIN ChIRP_probe19**	GACGAGAGAGGAGGGTGATA	ODD
**RAIN ChIRP_probe20**	CTTGGACCTTGGGATACTAA	EVEN

Supplementary Table 4 Clinical features of PTC patients selected for Nanostring analysis

Metastatic Samples (DM)						Control Samples (CTRL)					
**Sample**	**Age**	**Sex**	**pN**	**pT**	**pSTAGE**	**Sample**	**Age**	**Sex**	**pN**	**pT**	**pSTAGE**
DM33	51	F	1a	3	3	NO09	66	F	0	1b	1
DM30	47	M	1b	3	4c	NO13	51	F	0	3	3
DM55	82	F	1B	2	2	NO19	68	F	0	2	2
DM01	90	M	1b	4a	4a	NO26	22	M	0	1b	1
DM56	-	M	-	-	-	NO33	67	F	0	2	2
DM51	53	M	1B	3	4a	NO41	57	F	0	2	2
DM34	47	M	0	3	3	NO43	64	F	0	2	2
DM45	19	F	1b	3	2	NO44	59	F	0	1b	1
DM43	22	F	1a	4a	2	NO47	47	F	0	1b	1
DM06	73	M	1b	3	4a	NO53	-	M	0	2	-
DM28	59	F	1b	4a	4a	NO54	-	F	0	1b	-
DM29	58	F	0	3	3	NO57	-	F	0	1b	-
DM12	71	F	1a	4a	4a	CT29	30	M	0	1B	-
DM20	63	M	1b	3	4c	CT52	22	M	0	1A	-
DM04	80	M	1b	3	4a	NO15	57	F	0	1b	1
DM48	63	F	1a	4a	4a	NO20	53	F	0	1b	1
DM15	68	F	1b	3	4a	NO21	63	F	0	3	3
NI74	68	M	1a	3b	-	NO29	64	F	0	3	3
DM37	34	F	1a	3	1	NO32	64	F	0	1b	1
DM41	28	F	1b	3	2	NO35	54	F	0	1b	1
DM52	58	M	0	1b	1	NO36	61	F	0	1b	1
DM14	68	F	1a	3	3	NO46	47	F	0	1b	1
DM21	63	F	0	2	4a	NO58	-	F	0	1a	-
DM07	72	F	1a	3	4c	NO74	-	M	0	1n	-
