# Supplementary figures and images for: Exploring the transcriptional cooperation between RUNX2 and its associated elncRNA RAIN
Source: Cell Death Dis. 2024 Sep 14;15(9):673. doi: 10.1038/s41419-024-07058-x (PMC11399121; doi:10.1038/s41419-024-07058-x)

TPC1

MDA-T41

NT  
sgRNA

RAIN  
sgRNA

NT  
sgRNA

RAIN  
sgRNA

RUNX2

RUNX2

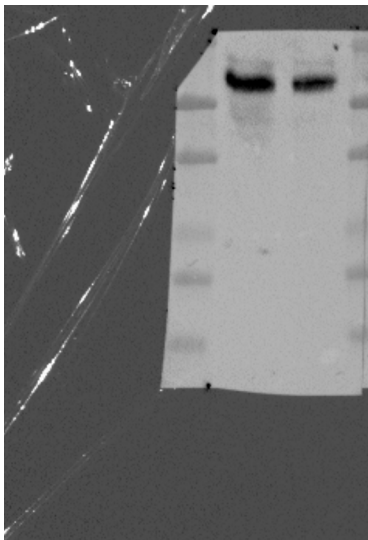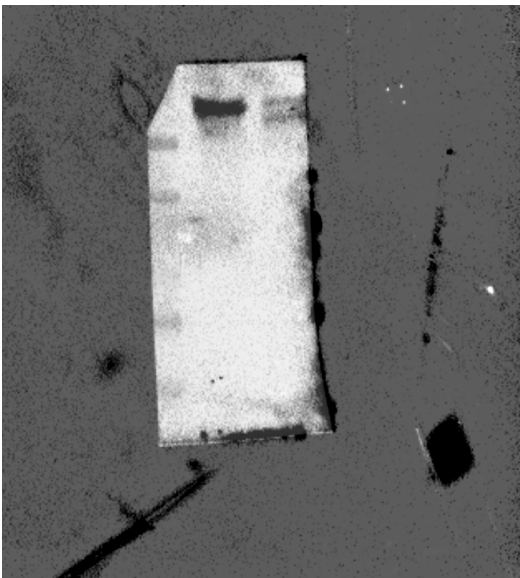

Actin

Actin

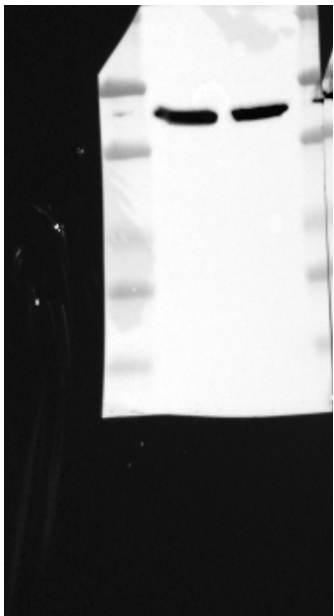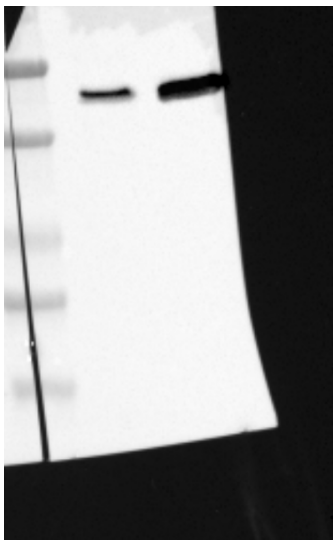

Supplement: Supplementary file 2 — Original Data [file 41419_2024_7058_MOESM2_ESM.pdf]
